# Use of ustekinumab for the treatment of pediatric keratosis lichenoides chronica: A novel therapeutic approach

**DOI:** 10.1016/j.jdcr.2025.09.007

**Published:** 2025-09-19

**Authors:** Lucie Joerg, Aimi Rothrock, Joseph Schwartz

**Affiliations:** a Albany Medical College, Albany, New York; b Department of Pathology and Laboratory Medicine, Albany Med Health System, Albany, New York; c J Schwartz MD, PLLC, Troy, New York

**Keywords:** keratosis lichenoides chronica, pediatric inflammatory dermatoses, rare disease, skin of color dermatology, ustekinumab

## Introduction

Keratosis lichenoides chronica (KLC), or Nekam’s disease, is a rare, progressive inflammatory skin disorder characterized by violaceous, hyperkeratotic papules or plaques typically arranged in a linear or reticular pattern.^1^^,^^2^ Cases affecting children are even more uncommon, with pediatric cases comprising roughly a quarter of reported KLC cases, with a mild male predominance.^3^ Furthermore, data on KLC in patients with skin of color remains very limited, underscoring a gap in the existing literature. KLC is usually slightly or nonpruritic with symmetrical lesions on the trunk and extremities.^3^ These manifestations are often accompanied by facial lesions bearing a resemblance to rosacea or seborrheic dermatitis.^1^ While the etiology of KLC is yet to be elucidated, germline mutations in the NLRP1 inflammasome sensor have been identified in familial cases showing congenital onset.^4^ Given its scarce prevalence and limited responsiveness to therapy, KLC currently lacks a standardized treatment protocol.^5^

## Case report

A 14-year-old Hispanic female with skin of color presented to the dermatology clinic for evaluation of pruritic facial, truncal, and bilateral extremity lesions. According to the family, the facial lesions developed shortly after birth and subsequently manifested on the trunk and extremities a few years later.

**Fig 1:**
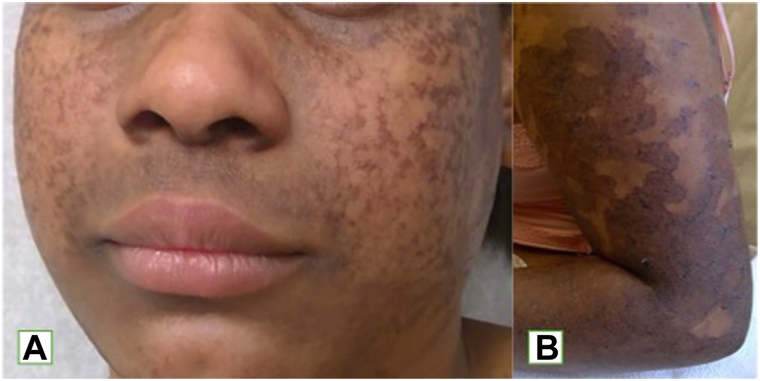
**A,** Reticulated hyperkeratotic plaques with associated pigmentary alteration on the face before treatment with ustekinumab. **B,** Left arm demonstrating hyperpigmentation, serpiginous scaling, and hyperkeratotic plaques before treatment with ustekinumab.

On examination, hyperpigmented and hyperkeratotic plaques with serpiginous scaling were noted in a reticular distribution overlying the aforementioned regions (Fig 1, *A* and *B*). The scalp, breasts, abdomen, groin, palms, and soles were spared. There was no evidence of nail pitting or subungual hyperkeratosis. She reported intermittent flares involving the arms and legs, with no identifiable triggering factors. Apart from pruritus, the patient denied any associated signs or symptoms. The patient's past medical history, which included microcephaly and developmental delay, as well as her review of systems and family history, was noncontributory.

**Fig 2:**
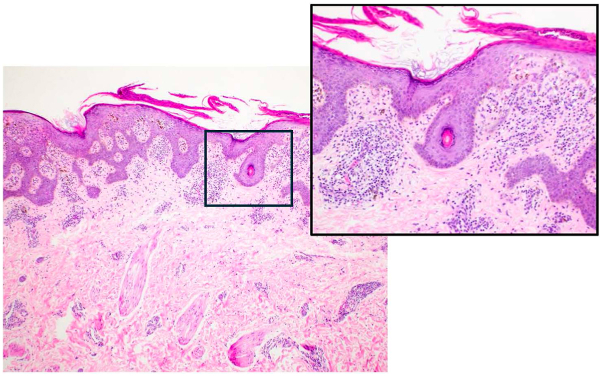
Hematoxylin and eosin-stained slide from the patient’s left posterior shoulder shave biopsy demonstrated foci of hyperkeratosis and parakeratosis with intracorneal neutrophilic microabscesses and a lichenoid tissue reaction with scattered dyskeratotic keratinocytes. Pigment incontinence and a mixed inflammatory infiltrate are seen within the superficial dermis.

The clinical differential diagnosis included psoriasis versus severe atopic dermatitis. Due to the unusual presentation, a biopsy was obtained at the initial visit. Histologic evaluation of a shave biopsy at the left posterior shoulder showed a lichenoid tissue reaction with epidermal hyperkeratosis, parakeratosis, variable acanthosis, and neutrophilic microabscesses in the stratum corneum. Conspicuous colloid bodies and dyskeratotic keratinocytes were noted, as well as a superficial dermal perivascular lymphohistiocytic infiltrate. Upon correlation with the clinical information provided, a diagnosis of KLC was established (Fig 2).

**Fig 3:**
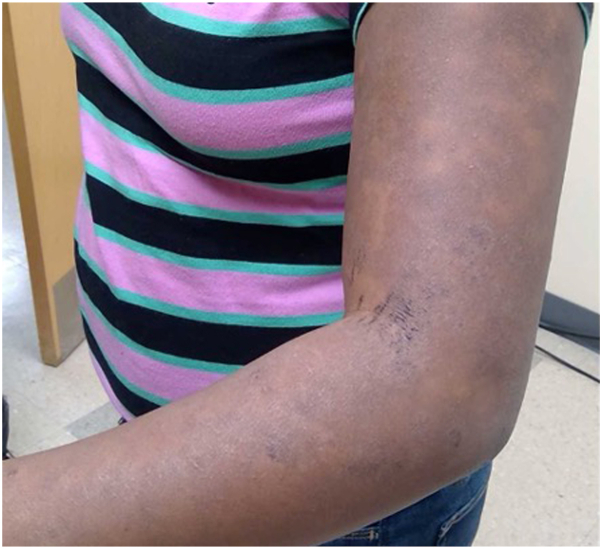
Left arm with reduced thickness and scale of plaques after the fourth injection of ustekinumab.

**Fig 4:**
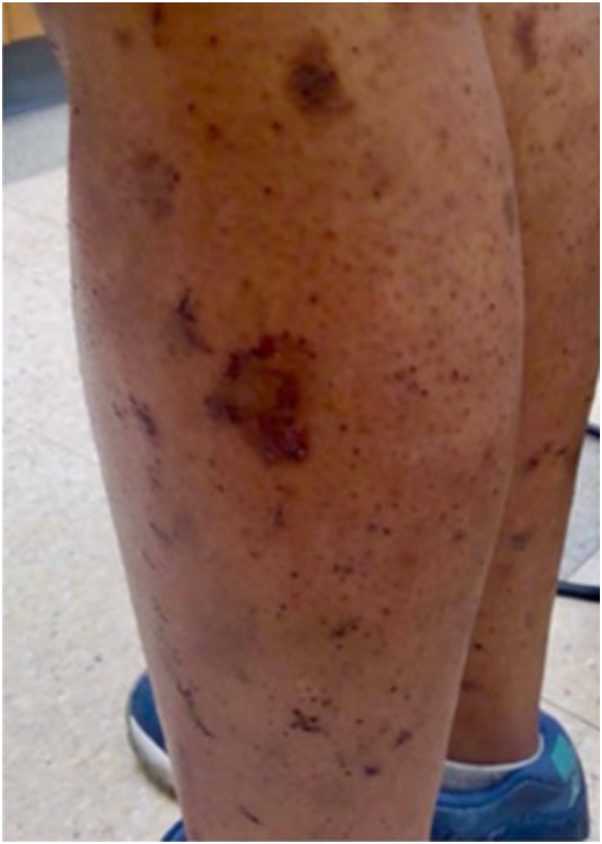
Legs demonstrating hyperpigmented and hyperkeratotic lesions returning approximately 6 months after the last dose of ustekinumab.

Treatment was started with topical steroids, including triamcinolone acetonide 0.1% cream and hydrocortisone 2.5% cream for 3 months, followed by tacrolimus 0.1% ointment for her facial lesions for 3 months. Narrowband UV-B phototherapy was added to the regimen for 3 months, with limited improvement in her lesions. Given the progression of her disease and suboptimal response to these therapies, an anti-inflammatory human monoclonal antibody therapy was recommended to the patient, and she was started on ustekinumab with standard psoriasis dosing of 45 mg subcutaneous injections (day 0, week 4, and every 12 weeks). Baseline laboratory evaluation, including complete blood count, comprehensive metabolic panel, tuberculosis screening, and serologic testing for hepatitis B, hepatitis C, and HIV, was unremarkable and remained negative throughout the course of ustekinumab therapy, supporting its safe use. After the fourth ustekinumab injection, excellent clearance of the rash was observed, with a marked reduction in the thickness and scale of her plaques (Fig 3). Postinflammatory hyperpigmentation persisted, especially on her face. Adverse effects, including cough, fever, fatigue, or injection site reaction, were not reported. The patient’s skin remained clear while on ustekinumab; however, due to a lack of compliance with office visits, follow-up doses were missed, and lesions returned approximately 6 months after the last dose of ustekinumab (Fig 4).

## Discussion

KLC represents a therapeutic challenge in pediatric and adult populations, with treatments typically based on symptomatic management, such as minimizing pruritus and control of hyperkeratosis. Given its extreme rarity and lack of consensus on treatment guidelines, management approaches remain individualized. Once a diagnosis is made, it is not uncommon for patients to be prescribed medications effective in treating psoriasis and lichen planus.^3^ Existing literature indicates that the most successful treatment options have been systemic retinoids and phototherapy, including sunlight exposure, narrowband UV-B, retinoids plus UV-B, psoralen UV-A, and retinoids plus psoralen UV-A therapy.^3^ In patients with darker skin tones, KLC may present with postinflammatory hyperpigmentation and minimal erythema, as seen in this patient, warranting tailored treatment considerations. Counseling individuals on potential pigmentary outcomes is essential for setting expectations and supporting adherence.

Although not considered a first-line treatment option for KLC, ustekinumab was prescribed as an alternative systemic agent approved for pediatric use. Ustekinumab is a human monoclonal antibody targeting the p40 subunit of IL-12 and IL-23.^6^ While not approved to treat KLC, ustekinumab’s efficacy in pediatric psoriasis, another chronic inflammatory dermatosis, provided a rationale for its off-label use in a patient unresponsive to other therapies. Phototherapy is not commonly reported in pediatric KLC, with many patients exhibiting clinical improvement or complete clearance following exposure to sunlight, especially in the summer.^3^ However, this patient did not respond to seasonal sunlight or UV-B phototherapy. Following the fourth 45 mg subcutaneous dose of ustekinumab (at approximately week 30 of treatment), the patient experienced significant clinical improvement with near-complete resolution of active lesions, supporting its potentially novel application in refractory pediatric KLC. Although generally infrequent and not observed in this patient, adverse effects of ustekinumab, such as nasopharyngitis, headache, and hypersensitivity reactions, should be monitored.^7^^,^^8^

Case reports remain critical for expanding our understanding of rare dermatoses like KLC, particularly when successful treatment is achieved with off-label immunomodulatory agents. In addition to our patient’s positive response to ustekinumab, several reports have documented favorable outcomes with other off-label therapies. Janus kinase inhibitors such as upadacitinib and monoclonal antibody therapies like efalizumab (withdrawn from the market in 2009) have shown efficacy in refractory KLC.^9^^,^^10^ These reports, though limited, underscore the therapeutic potential of targeted agents in modulating the inflammatory pathways implicated in KLC pathogenesis. Notably, the absence of randomized trials and standardized treatment protocols highlights a significant gap in therapeutic guidance for both pediatric and adult KLC. Given the rarity of this condition, case-based evidence remains essential to guide clinical decision-making and inform future research directions.

## Conflicts of interest

Dr Schwartz is on the speakers' bureau for Janssen Biotech, Inc, and has received honoraria for speaking engagements, travel support for related presentations, and meals from a Janssen sales representative. Drs Joerg and Rothrock have no conflicts of interest to declare.
